# Comparison of Glucose Tolerance between Kidney Transplant Recipients and Healthy Controls

**DOI:** 10.3390/jcm8070920

**Published:** 2019-06-27

**Authors:** Hisao Shimada, Junji Uchida, Shunji Nishide, Kazuya Kabei, Akihiro Kosoku, Keiko Maeda, Tomoaki Iwai, Toshihide Naganuma, Yoshiaki Takemoto, Tatsuya Nakatani

**Affiliations:** 1 Department of Urology, Osaka City University Graduate School of Medicine, Osaka 545-8585, Japan; 2 Department of Nursing, Osaka City University Hospital, Osaka 545-8585, Japan

**Keywords:** kidney transplantation, glucose intolerance, insulin secretion, insulin resistance, oral glucose tolerance test, healthy subject

## Abstract

Post-transplant hyperglycemia and new-onset diabetes mellitus after transplantation (NODAT) are common and important metabolic complications. Decreased insulin secretion and increased insulin resistance are important to the pathophysiologic mechanism behind NODAT. However, the progression of glucose intolerance diagnosed late after kidney transplantation remains clearly unknown. Enrolled in this study were 94 kidney transplant recipients and 134 kidney transplant donors, as the healthy controls, who were treated at our institution. The 75 g-oral glucose tolerance test (OGTT) was performed in the recipients, and the healthy controls received an OGTT before donor nephrectomy. We assessed the prevalence of glucose intolerance including impaired fasting glucose and/or impaired glucose tolerance, as well as insulin secretion and insulin resistance using the homeostasis model assessment, and compared the results between the two groups. Multivariate analysis after adjustment for age, gender, body mass index, estimated glomerular filtration rate, and systolic blood pressure showed that the prevalence of glucose intolerance, insulin resistance, insulin secretion, and 2 h plasma glucose levels were significantly higher in the kidney transplant recipients compared to the healthy controls. Elevation of insulin secretion in kidney transplant recipients may be compensatory for increase of insulin resistance. Impaired compensatory pancreas β cell function may lead to glucose intolerance and NODAT in the future.

## 1. Introduction

Post-transplant hyperglycemia and new-onset diabetes mellitus after transplantation (NODAT) are common and significant metabolic complications in kidney transplant recipients (KTRs) which can lead to increased mortality and cardiovascular morbidity [1,2,3]. Similar to type 2 diabetes mellitus (DM), decreased insulin secretion and increased insulin resistance are important to the pathophysiologic mechanism behind NODAT [4]. Previous reports have shown that impaired insulin secretion is a more dominant mechanism for the development of NODAT compared to type 2 DM [5]. However, the exact mechanism of glucose intolerance diagnosed late after kidney transplantation remains unknown [6], although immunosuppressive agents such as calcineurin inhibitors, steroids, and mammalian target of rapamycin inhibitors are thought to cause glucose intolerance following kidney transplantation [1,2,3]. The increased prevalence of cardiovascular events in transplant recipients is an issue that remains to be solved. Abnormal glucose homeostasis is considered to be one of the established risk factors for the development of cardiovascular events following kidney transplantation [7], but there have been few reports comparing glucose tolerance between KTRs and healthy subjects.

The oral glucose tolerance test (OGTT) has many advantages over fasting plasma glucose for diagnosing glucose intolerance, as it not only accurately identifies persons with DM but also identifies those with impaired fasting glucose (IFG) and impaired glucose tolerance (IGT). Abnormal glucose tolerance determined by the OGTT is a risk factor for the future development of type 2 DM in general populations [8,9], and the OGTT has also been established as a sensitive tool to detect NODAT and glucose intolerance in KTRs [10,11]. The homeostasis model assessment (HOMA) model is a well-known method used for the quantitative verification of insulin resistance and insulin secretion. In this model, fasting glucose levels and fasting insulin levels are mainly defined by feedback of glucose release from the liver and insulin secretion from pancreatic β cells [12]. The aim of this study was to compare glucose tolerance between KTRs and healthy subjects using the OGTT, and we assessed the prevalence of glucose intolerance including IFG and/or IGT as well as insulin secretion and insulin resistance using the HOMA. The correct homeostasis model assessment evaluation using a computer program was reported to be another verification of insulin sensitivity and pancreatic β cell function [13]. However, we used the homeostasis model assessment of insulin resistance (HOMA-R) and homeostasis model assessment of β cell function (HOMA-β) in this study, because we had previously reported on glucose intolerance in kidney transplant recipients using these methods [11].

## 2. Patients and Methods

### 2.1. Study Design and Participants

This study was a single-center, cross-sectional, observational investigation conducted at Osaka City University Graduate School of Medicine. Ninety-four out of 101 KTRs who underwent a transplant at our institution and consented to participate in this study were enrolled (KTR group) (Figure S1). To compare glucose tolerance between KTRs and healthy subjects, 134 kidney transplant donors who underwent a nephrectomy at our institution between 2006 and 2017 were enrolled in this study as the healthy controls (HC group). A 75 g-OGTT was performed in the KTR group from October 2010 to April 2012, and the HC group received a 75 g-OGTT before donor nephrectomy. For the KTR group, the inclusion criteria were as follows: (1) stable calcineurin inhibitor (CNI) levels for the last 6 months, (2) no prior evidence of DM, (3) at least a year after transplantation, (4) serum creatinine below 2.0 mg/dL, and (5) stable renal function for the last 6 months. The following recipients were excluded from this study: (1) patients with acute infection, liver dysfunction, abnormal thyroid tests, history of gastrectomy, or chronic pancreatitis and (2) patients who had histories of hepatitis B virus and/or hepatitis C virus infection irrespective of being treated or untreated. This study was approved by the Ethics Committee of Osaka City University Graduate School of Medicine (No. 4120). We provided patients with information explaining the proposed research plan (the purpose, required individual data, and duration of research) by means of an information website of our hospital and gave them the opportunity to opt out, and all the procedures were in accordance with the Helsinki Declaration of 2000 and the Declaration of Istanbul 2008.

### 2.2. Immunosuppressive Regimen 

Standard immunosuppressive regimen for kidney transplantation consisted of basiliximab (BAS), methylprednisolone (MP), CNI (cyclosporine or tacrolimus), and antimetabolites (mycophenolate mofetil or mizoribine or azathioprine). CNI and antimetabolites were initiated 3 days before transplantation. BAS has been administered in all recipients since 2002 at a dose of 20 mg/day at the time of transplantation and 4 days after transplantation. MP was intravenously administered at a dose of 500 mg at the time of transplantation and orally administered at 40 mg/day 1 to 7 days after transplantation, the dose of which was decreased to 24, 12, 8, and 4 mg/day every week. For treatment of acute cellular rejection events, MP was intravenously administered at a dose of 500 mg/day for 3 days alone or in combination with deoxyspergualin (5 mg/kg/day: 5–7 days).

### 2.3. Data Collection 

Patient characteristics (age, gender) and clinical data [estimated glomerular filtration rate (eGFR), fasting plasma glucose (FPG), fasting immunoreactive insulin, hemoglobin A1c, triglyceride, total cholesterol, high-density lipoprotein-cholesterol, low-density lipoprotein-cholesterol] were collected from electronic medical records in all subjects enrolled in this study. eGFR was estimated by the modified Modification of Diet in Renal Disease Equation using the Japanese coefficient [14]. Blood samples were obtained after overnight fasting. Body mass index (BMI) was calculated as body weight in kilograms divided by the square of body height in meters (kg/m^2^). Blood pressure was reported as the average of five automated measurements taken at 3-min intervals. Clinical parameters of the KTR group such as type of CNIs, donor type, and post-transplant duration were collected. Dyslipidemia was defined by oral administration such as statin and polyunsaturated fatty acid, triglycerides over 150 mg/dL, or HDL cholesterol below 40 mg/dL, or LDL cholesterol over 140 mg/dL. Hypertension was defined by administration of antihypertensive drug, systolic blood pressure over 140 mmHg, or diastolic blood pressure over 90 mmHg. The administration of angiotensin converting enzyme inhibitor and/or angiotensin II receptor blocker, β-blocker, calcium channel blocker, loop diuretic, and thiazide diuretic were evaluated in all subjects. 

### 2.4. Glucose Intolerance, Insulin Resistance, and β Cell Function

All patients underwent an OGTT in the morning after overnight fasting. Blood samples were drawn for determining plasma glucose and insulin before glucose loading and at 30 and 120 min after glucose loading. According to the World Health Organization [15], normal glucose tolerance (NGT) was defined as FPG and 2-h plasma glucose of <110 mg/dL and <140 mg/dL, IFG as 110–126 mg/dL and <140 mg/dL, IFG/IGT as 110–126 mg/dL and 140–200 mg/dL, and DM as ≥ 126 mg/dL and/or ≥200 mg/dL, respectively. Glucose intolerance consisted of IFG, IGT, IFG/IGT, and DM. Insulin resistance was estimated using the HOMA of insulin resistance (HOMA-R) according to the formula HOMA-R = fasting insulin (mU/L) × FPG (mg/dL)/405. For the assessment of pancreatic β cell function, we used the HOMA of β cell function (HOMA-β) according to the formula HOMA-β = 360 × fasting insulin (mU/L)/(FPG (mg/dL)-63), and the insulinogenic index = (insulin 30 min-fasting insulin (mU/L))/(plasma glucose 30 min-FPG (mg/dL)) [12].

### 2.5. Statistical Analysis

Categorical variables were expressed as count and percentage, and continuous variables were expressed as mean ± standard deviation, or median and interquartile range, or range. Differences between the groups were examined by Student’s t-test or Mann-Whitney U-test. Categorical variables were compared using chi-squared analysis. Logistic regression analysis was used to test the influence of KTR group or HC group on the glucose intolerance (versus normal glucose tolerance). We also performed logistic regression analysis adjusted for multiple models (Model 1: Adjusted for age, gender, and BMI; Model 2: Adjusted for Model 1 and eGFR; Model 3: Adjusted for Model 2 and SBP). Linear regression analysis was used to test if the group (KTR group or HC group) was related to various dependent variables. We also performed linear regression analysis adjusted for multiple models (Model 1: Adjusted for age, gender, and BMI; Model 2: Adjusted for Model 1 and eGFR; Model 3: Adjusted for Model 2 and SBP). All statistical analyses were performed with SPSS version 22.0 for windows (IBM Japan, Tokyo, Japan). A *p*-value of less than 0.05 was considered statistically significant.

## 3. Results

### 3.1. Study Participants

The comparison of characteristics between the KTR and HC groups is presented in Table 1. The age in the KTR group was significantly younger than that in the HC group, while BMI in the HC group was significantly higher than that in the KTR group. Hemoglobin A1c levels in the KTR group were lower than those in the HC group. eGFR was lower in the KTR group compared to the HC group. The prevalence of hypertension in the KTR group was significantly higher than that in the HC group. The clinical parameters related to kidney transplantation in the KTR group are shown in Supplementary Table S1.

### 3.2. Insulin Secretion and Resistance in Subjects with NGT and Glucose Intolerance

In the KTR group, NGT was detected in 74 (78.7%) patients, while 20 (21.3%) had glucose intolerance including two with IFG, 10 with IGT, two with IFG/IGT, and six with DM. Meanwhile, in the HC group, NGT was detected in 107 (79.9%) patients, while 27 (20.1%) had glucose intolerance including four with IFG, 16 with IGT, three with IFG/IGT, and four with DM. There was no significant difference in the prevalence of glucose intolerance between the two groups. HOMA-R in the KTR group tended to be higher than that in the HC group (*p* = 0.051). HOMA-β in the KTR group was significantly higher than that in the HC group. There was also no significant change in the insulinogenic index between the two groups. There were no significant changes in FPG and 2 h plasma glucose levels between the two groups (Table 2).

### 3.3. Comparison of Prevalence of Glucose Intolerance by Multivariate Logistic Regression Analysis

Multivariate logistic regression analysis was performed to compare the prevalence of glucose intolerance between the two groups (Table 3). There was no significant association in the unadjusted Model and Model 1 (adjusted for age, gender, and BMI). In Model 2 (adjusted for Model 1 and eGFR), there was a statistically significant association between glucose intolerance and group (KTR group versus HC group) (OR = 3.544, 95% CI = 1.143–10.986, *p* = 0.028). In Model 3 (adjusted for Model 2 and SBP), there was a statistically significant association between glucose intolerance and group (KTR group versus HC group) (OR = 3.794, 95% CI = 1.200–11.996, *p* = 0.023).

### 3.4. Comparison of FPG and 2 h Plasma Glucose Levels by Multivariate Logistic Regression Analysis

The results of linear regression analysis of FPG and 2 h plasma glucose levels are shown in Table 4. In all models, there was no significant association between FPG and group (KTR group versus HC group). However, in Model 1, Model 2, and Model 3, there was a statistically significant association between 2 h plasma glucose levels and group (KTR group versus HC group) (Model 1: B = 10.713, S.E. = 4.861, *p* = 0.029; Model 2: B = 15.079, S.E. = 7.311, *p* = 0.040; Model 3: B = 15.091, S.E. = 7.329, *p* = 0.041). 

### 3.5. Comparison of HOMA-R and HOMA-β by Multivariate Logistic Regression Analysis

Table 5 shows the results of linear regression analysis of HOMA-R and HOMA-β. In Model 1, Model 2, and Model 3, there was a statistically significant association between HOMA-R and group (KTR group versus HC group) (Model 1: B = 0.516, S.E. = 0.170, *p* = 0.003; Model 2: B = 0.615, S.E. = 0.256, *p* = 0.017; Model 3: B = 0.616, S.E. = 0.256, *p* = 0.017). In all models, there was a statistically significant association between HOMA-β and group (KTR group versus HC group) (unadjusted Model: B = 15.850, S.E. = 6.341; *p* = 0.013; Model 1: B = 24.581, S.E. = 6.417, *p* < 0.001; Model 2: B = 28.699, S.E. = 9.658, *p* = 0.003; Model 3: B = 28.715, S.E. = 9.689, *p* = 0.003). 

## 4. Discussion

In this study, multivariate regression analysis revealed that the prevalence of glucose intolerance in the KTR group was significantly higher than in the HC group. Moreover, insulin resistance in the KTR group was significantly higher than that in the HC group, and insulin secretion in the KTR group was also higher than that in the HC group. The elevation of insulin secretion may be compensatory for the increase of insulin resistance in the KTR group. To our knowledge, this is the first demonstration comparing glucose tolerance between KTRs and healthy subjects. 

The pathophysiology of NODAT is similar to type 2 DM but with important differences. Previous reports have shown that the primary pathophysiological defect is more pancreatic β cell dysfunction in NODAT compared to type 2 DM [5]. However, the mechanism of glucose intolerance diagnosed late after kidney transplantation is not clear [6]. Because of the prolonged and elevated insulin resistance due to immunosuppressive agents such as steroids, CNIs, and mTOR inhibitors administered for a long time at a late post-transplant stage, long-term compensatory insulin secretion of pancreatic β cells may be required to prevent impaired glucose metabolism in KTRs. Additional risk factors of DM such as gaining weight after transplantation or aging may lead to the collapse of pancreatic β cells function in these patients. As a consequence, glucose intolerance and DM in KTRs may occur at a late post-transplant stage despite a concomitant decrease in steroid use and CNI blood levels.

It has been established that IGT and IFG are risk factors for developing type 2 DM in the future in general populations [6,7]. In our study, multivariate logistic regression analysis revealed that the prevalence of glucose intolerance based on the OGTT such as IFG, IGT, and DM in the KTR group was higher than that in the HC group. KTRs may therefore have a higher risk for new-onset DM compared to healthy subjects. One study reported that the occurrence of acute rejection and NODAT within the first post-transplantation year showed a similar impact on long-term transplant survival. Moreover, NODAT seems to be associated with death with a functioning graft [16]. As pre-stages of NODAT, IFG and IGT have been introduced as gluco-metabolic targets in an effort to reduce the risk of developing chronic transplant-associated morbidity and mortality by implementing proper management approaches during pre-and post-transplant stages [17]. Assessment of glucose intolerance based on the OGTT may also be more important for achieving excellent transplant outcomes in KTRs.

KTRs have an increased risk of premature death due to cardiovascular disease, malignancy, and infectious disease, as they are the predominant causes of mortality [18]. Immunosuppressive therapy may potentiate these risk factors. However, the increased long-term mortality after kidney transplantation cannot be fully explained by this. Hyperglycemia is reported to be a risk marker for cardiovascular disease and cancer among healthy subjects without DM [19,20]. In a previous study, 2-h post-load glucose concentrations indicated a risk of all cause and cardiovascular morbidity in a general population without known DM [21]. Multivariate regression analysis in our study identified that 2-h plasma glucose levels in the KTR group were higher than those in the HC group. Increased 2-h plasma glucose levels in KTRs may elevate the risk of all cause and cardiovascular morbidity, and KTRs may tend to have subclinical hyperglycemia. 

This study has several important limitations. Because it was a cross-sectional, observational study, the risk for glucose intolerance was not completely clarified. Also, the risk factor data of NODAT, such as family history of DM and hypomagnesemia were not available [22,23]. However, this may be the first demonstration comparing glucose tolerance between KTRs and healthy subjects. Our study may be helpful for understanding the status of glucose tolerance in KTRs receiving immunosuppressive therapy. Prospective and cohort studies involving a much larger population are needed to identify the mechanism of glucose intolerance in KTRs.

## 5. Conclusions

In conclusion, insulin resistance as well as insulin secretion in the KTR group were significantly higher than those in the HC group. Moreover, the prevalence of glucose intolerance and 2-h plasma glucose levels in the KTR group were significantly higher than those in the HC group. The elevation of insulin secretion may be compensatory for the increase of insulin resistance in KTRs. In these recipients, the impaired compensatory pancreas β cell function may lead to NODAT and glucose intolerance late after kidney transplantation.

## Figures and Tables

**Table 1:** Clinical characteristics of kidney transplant recipients and healthy controls.

Variables	KTR Group	HC Group	*p*
	Median [IQR] or %	Median [IQR] or %	
	*n* = 94	*n* = 134	
Age (years)	47 (37, 58)	57 (49, 65)	<0.001
Male gender (%)	48.9	39.6	0.176
Body mass index (kg/m^2^)	20.36 (18.52, 22.52)	22.36 (20.74, 24.61)	<0.001
Serum creatinine (mg/dL)	1.21 (0.93, 1.43)	0.68 (0.59, 0.79)	<0.001
eGFR (mL/min/1.73 m^2^)	47.02 (40.69, 55.65)	78.08 (69.93, 87.55)	<0.001
Hemoglobin (g/dL)	12.1 (11.1, 13.2)	13.8 (13.0, 14.8)	<0.001
Hematocrit (%)	36.35 (34.03, 39.08)	41.35 (39.23, 43.95)	<0.001
HbA1c (%)	5.4 (5.2, 5.8)	5.7 (5.5, 5.9)	<0.001
Triglycerides (mg/dL)	99 (74, 137)	94 (69, 138)	0.823
Total cholesterol (mg/dL)	197 (179, 217)	202 (181, 223)	0.231
HDL cholesterol (mg/dL)	66 (57, 74)	61 (50, 72)	0.077
LDL cholesterol (mg/dL)	111 (92, 128)	116 (101, 138)	0.036
Dyslipidemia (%)	38.3	43.3	0.494
Systolic blood pressure (mmHg)	120 (112, 126)	117 (106, 131)	0.478
Diastolic blood pressure (mmHg)	74 (68, 80)	71 (64, 78)	0.127
Hypertension (%)	76.6	15.7	<0.001
Administration of ARB (%)	53.2	12.7	<0.001
Administration of ACEi (%)	23.6	0.7	<0.001
Administration of β-blocker (%)	8.5	0	<0.001
Administration of calcium channel blocker (%)	44.7	8.2	<0.001
Administration of thiazide diuretics (%)	5.3	0.7	0.084
Administration of loop diuretics (%)	3.2	0	0.069
Post-transplant duration (years)	5.4 (2.8, 9.6)	-	-
Donor type (cadaver) (%)	16.0	-	-
CNI (Tacrolimus) (%)	39.4	-	-

KTR, kidney transplant recipients; HC, healthy controls; BMI, body mass index; eGFR, estimated glomerular filtration rate; HDL cholesterol, high-density lipoprotein cholesterol; LDL cholesterol, low-density lipoprotein cholesterol; ARB, angiotensin II receptor blocker; ACEi, angiotensin converting enzyme inhibitor; CNI, calcineurin inhibitors.

**Table 2:** Glucose intolerance between kidney transplant recipients and healthy controls.

Variables	KTR Group	HC Group	*p*
	Median [IQR] or %	Median [IQR] or %	
	*n* = 94	*n* = 134	
Fasting plasma glucose (mg/dL)	95 (88, 99)	95 (90, 101)	0.471
2 h plasma glucose (mg/dL)	113 (96, 132)	114 (96, 129)	0.911
Fasting IRI (μU/mL)	6.5 (5.3, 8.7)	5.6 (4.0, 8.4)	0.027
Glucose intolerance (%)	19.4	20.1	1.000
HOMA-R (mIU/mmol L-2)	1.59 (1.15, 1.98)	1.37 (0.89, 2.00)	0.051
HOMA-β (mIU/mmol)	73.78 (53.65, 105.65)	64.02 (47.25, 92.59)	0.027
Insulinogenic Index (μU 10/mg)	0.80 (0.49, 1.22)	0.78 (0.39, 1.26)	0.804

KTR, kidney transplant recipients; HC, healthy controls; IRI, immunoreactive insulin; HOMA-R, homeostasis model assessment of insulin resistance; HOMA-β, homeostasis model assessment of β cell function. IQR, interquartile range.

**Table 3:** Multiple logistic regression analysis for prevalence of glucose intolerance (glucose intolerance versus normal glucose tolerance) between kidney transplant recipients and healthy controls.

	OR	95% CI	*p*
Unadjusted Model: KTR (vs. HC)	0.939	0.483, 1.825	0.852
Model 1: KTR (vs. HC) adjusted for age, gender, and BMI	1.374	0.645, 2.927	0.410
Model 2: KTR (vs. HC) adjusted for Model 1 and eGFR	3.544	1.143, 10.986	0.028
Model 3: KTR (vs. HC) adjusted for Model 2 and SBP	3.794	1.200, 11.996	0.023

KTR, kidney transplant recipients; HC, healthy controls; BMI, body mass index; eGFR, estimated glomerular filtration rate; SBP, systolic blood pressure; OR, odds ratio; 95% CI, 95% confidence interval.

**Table 4:** Correlation between fasting plasma glucose and 2 h plasma glucose with presence of kidney transplantation in adjusted linear regression analysis.

	FPG			2-hPG		
	B	S.E.	*p*	B	S.E.	*p*
Unadjusted Model: KTR (vs. HC)	–0.349	1.439	0.809	2.101	4.604	0.649
Model 1: KTR (vs. HC) adjusted for age, gender, and BMI	1.254	1.488	0.400	10.713	4.861	0.029
Model 2: KTR (vs. HC) adjusted for Model 1 and eGFR	4.062	2.226	0.069	15.079	7.311	0.040
Model 3: KTR (vs. HC) adjusted for Model 2 and SBP	4.068	2.229	0.069	15.091	7.329	0.041

FPG, fasting plasma glucose; 2-hPG, 2-h plasma glucose; KTR, kidney transplant recipients; HC, healthy controls; BMI, body mass index; eGFR, estimated glomerular filtration rate; SBP, systolic blood pressure; B, coefficient estimate; S.E., standard error.

**Table 5:** Correlation between HOMA-R and HOMA-β with presence of kidney transplantation in adjusted linear regression analysis.

	HOMA-R			HOMA-β		
	B	S.E.	*p*	B	S.E.	*p*
Unadjusted Model: KTR (vs. HC)	0.205	0.170	0.229	15.850	6.341	0.013
Model 1: KTR (vs. HC) adjusted for age, gender, and BMI	0.516	0.170	0.003	24.581	6.417	<0.001
Model 2: KTR (vs. HC) adjusted for Model 1 and eGFR	0.615	0.256	0.017	28.699	9.658	0.003
Model 3: KTR (vs. HC) adjusted for Model 2 and SBP	0.616	0.256	0.017	28.715	9.689	0.003

HOMA-R, homeostasis model assessment of insulin resistance; HOMA-β, homeostasis model assessment of β cell function; KTR, kidney transplant recipients; HC, healthy controls; BMI, body mass index; eGFR, estimated glomerular filtration rate; SBP, systolic blood pressure; B, coefficient estimate; S.E., standard error.

## Supplementary Materials

The following are available online at https://www.mdpi.com/2077-0383/8/7/920/s1, Figure S1: Flow chart of patients’ enrollment, Table S1: Clinical parameters of kidney transplant recipients.
