# Supplementary material for: Comparison of Glucose Tolerance between Kidney Transplant Recipients and Healthy Controls
Source: J Clin Med. 2019 Jun 27;8(7):920. doi: 10.3390/jcm8070920 (PMC6678426; doi:10.3390/jcm8070920)
Supplement: Supplementary file 1 [file jcm-08-00920-s001.pdf]

Supplementary Materials:

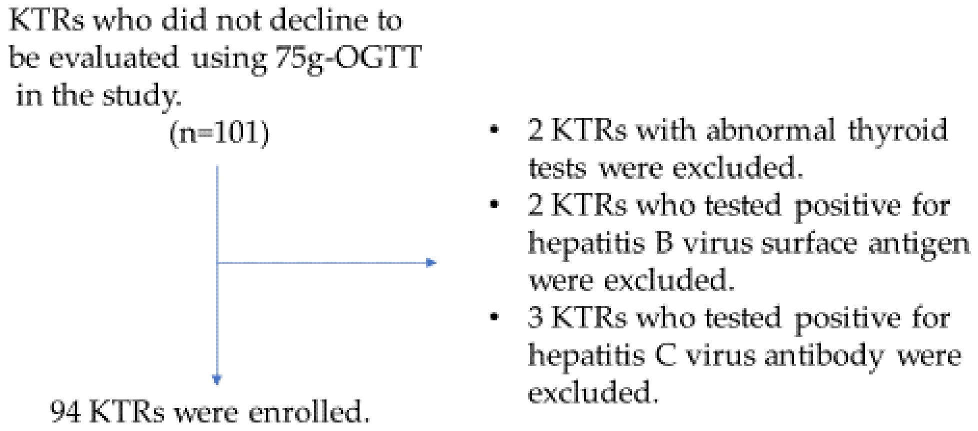

Supplementary Figure 1. Flow chart of patients' enrollment.

Supplementary Table 1. Clinical parameters of kidney transplant recipients.

Variables	KTR group<br>Median [IQR] or %<br>n = 94
Dialysis duration (years)	2.3 [0.8,6.6]
Weight gain after KTx (kg)	1.0 [-2.5,3.0]
Vitamin D deficiency (%)	3.2
High pre-KTx triglyceride concentration (%)	16.0
Hyperuricaemia (%)	33.0
Acute rejection (%)	23.4
CMV infection (%)	27.7
Cause of end-stage renal disease	
Chronic glomerulonephritis (%)	39.4
IgA nephropathy (%)	13.8
Nephrosclerosis (%)	3.2
ADPKD (%)	3.2
Other or unknown (%)	40.4
Immunosuppression	
Methylprednisolone (%)	96.8
Mycophenolate mofetil (%)	84.0
Mizoribine (%)	8.5
Azathioprine (%)	7.5
Everolimus (%)	0

KTR, kidney transplant recipients; KTx, kidney transplantation; CMV, cytomegalovirus; ADPKD, autosomal dominant polycystic kidney disease; IQR, interquartile range.
